# Odor Modulates Hand Movements in a Reach-to-Grasp Task

**DOI:** 10.3389/fnins.2020.00560

**Published:** 2020-06-16

**Authors:** Yang Yang, Xiaochun Wang

**Affiliations:** School of Psychology, Shanghai University of Sport, Shanghai, China

**Keywords:** odor, visual feedback, hand movement, multisensory integration, kinematics

## Abstract

Recent evidence suggests that target-relevant sensory stimuli (i.e., visual, auditory, and olfactory) can play important roles in the motor system. However, little is known about the effects of olfactory information on reaching and grasping movements. To determine whether odor stimuli affect hand movements, the reaching and grasping kinematic characteristics of 29 human participants were recorded using a three-dimensional video motion capture system. Participants received an odor stimulus by Sniffin’ Sticks and then reached toward and grasped a target. Grasping targets were apple, orange, ginger, and garlic. The odor stimulus was congruent with the target. The size of the odor-cued object (OCO) was the same size, smaller, or larger than a target to be grasped; or participants received odorless air while they viewed that target. They reached the target with one of two grips: a precision grip for a small target or a power grip for a larger target. The visual feedback was lost in half of 80 total trials after a start signal. It was no longer visible when participants reached the target. The results of repeated-measures analyses of variance followed by simple-effects analyses showed that when the size of the hand movement evoked by the odor cue was congruent with the size of the target, either both small or both large, the reaction time was significantly shorter than it was for odorless air. When participants received visual feedback throughout the trial, movement duration was significantly shorter if the odor cue was congruent with the size of the target or if odorless air was dispensed. When the size of hand movement evoked by the odor cue was incongruent with the size of the target, an interference effect was apparent on the maximum aperture time. The result of odorless air control group in a closed loop was shorter than incongruent odor group. In addition, visual feedback influenced the results such that the maximum aperture time occurred later when visibility was blocked only in the odorless air control condition. These results suggest that olfactory information has a positive effect on reach-to-grasp hand movements and that vision and olfaction may interact to optimize motor behavior.

## Introduction

In a sensorimotor control system, sensory information affects body movement. Complex environments require humans to have good perceptive functions providing many details (Castiello, 2005). Several studies have revealed that visual, auditory, and olfactory systems (Castiello et al., 2006; Tubaldi et al., 2008) play important roles in the sensorimotor system. In the visual system’s regulation of the sensorimotor system, there is a distinction between perception and behavior. In other words, both perception of visual information and execution of motor control are involved. Schmidt and Lee explained two different loops and discussed the role of feedback regulation (Schmidt and Lee, 2005). In the motor skill learning and controlling procedure, an actuator can receive sensory information and send to an effector, which is called open loop. A closed loop can correct the errors by visual feedback. There is a controller comparing the difference between the ideal and actual results. Woodworth divided movement procedure into two parts, namely, motor plan and control (Woodworth, 1970). In the planning phase, it created motor plan before actual movement and was helpful to activate movement. In the controlling phase, it could correct the movement error in time. According to the planning-control model (Glover and Dixon, 2001), in humans, the generation of motor planning depends on the cues around the target, whereas planning does not adjust or control behavior once the behavior is initiated. Previous research has focused primarily on vision as the dominant sensory modality that affects other sensory modalities. However, when a multisensory system exists, that is, when numerous sensory modalities are available and integrated by the central nervous system, a perceptual experience may be generated in the absence of a given sensory modality as other modalities become enhanced, an effect known as inverse effectiveness (Stein and Stanford, 2008). For example, a cup of white wine but colored red was described as a cup of red wine by tasters (Morrot et al., 2001). To a certain extent, it reflected the superiority of vision in the integration of vision and olfactory. Not only that, but several previous studies have found that the visual characteristics of the object, such as color and shape, can affect the individual’s olfactory functions of detecting, discriminating, and recognizing (Jay and Dolan, 2003; De Araujo et al., 2005; Dematte et al., 2008; Robert et al., 2010). When visual information is ambiguous, olfactory information can modulate the visual modality. It has been shown that olfactory information can modulate visual attention to point participants to a target that is congruent with the olfactory information (Chen et al., 2013). In addition, odor cues can evoke a perceptual change when a human is judging an unclear movement of a target (Kuang and Tao, 2014). They investigated how odors affect the direction of movement. When the directional perception was ambiguous, the olfactory information was significantly related to it and integrated the effects with visual information.

An odor stimulus can also be a cue that enters the processing system and influences motor behavior in humans. For example, during performing a word recognition task in a room scented with air fresher, the response to words such as cleaning and tidying up was faster than to that of other non-cleaning-related words (Holland et al., 2005). In addition, participants who were given biscuits as a reward cleaned a room more frequently than the control group that did not receive biscuits. When researchers investigated customer motivation and behavior, they concluded that providing certain ambient odors increases the time customers spend in a store (Vinitzky and Mazursky, 2011). A pleasant smell in a space will provide a better evaluation and memory of an experience than a bad smell (Cirrincione et al., 2014; Doucé et al., 2014). Compared with providing odorless air, diffusing pleasant odors, such as orange, seawater, or mint, also produces similar results (Schifferstein et al., 2011). Thus, several lines of evidence have suggested that olfaction can influence human planning and behavior.

Reach-to-grasp movements are typical for humans to handle objects. Such movements assist the human body in interacting with the environment during early childhood and in establishing perception of past experience for adults. For example, babies can grasp an apple, and we can lift a dumbbell. Hence, how humans modulate hand movements on the basis of sensory information is important. Most previous studies focused on the effects of semantic perception and quantity perception on grasping movement. Glover put a *grape* or *apple* label on the target and asked participants to grasp it. In the early stage of a grasping movement, the size of the grip aperture when grasping the *apple* object was larger than that when grasping the *grape* target, but as the distance between the hand and the target shortened, visual feedback adjusted this difference until it disappeared (Glover et al., 2004). Humans need to collect information about related properties, spatial position, and so on to complete a reach-to-grasp movement. One property that influences information collection is olfactory information, with different odor cues affecting the reaction and movement velocity of humans to a target. For example, representations evoked by olfactory information have been demonstrated to affect the size of a hand grip (Castiello et al., 2006). When an odor cue represents an object larger than the target to be grasped, the amplitude of the peak grip aperture is greater and the movement duration (MD) is longer than when the odor cue represents an object that is small (Parma et al., 2011). Conversely, when the odor cue represents a small object, the amplitude of the peak grip aperture for a large target to be grasped is smaller and the MD is shorter but the movement time is longer. Only when the odor cue represents a small object and the target to be grasped is also small was the hand movement planning reaction promoted. There is no difference when the odor cue represents a large object. When a target is large and an odor cue represents a small object, the maximum aperture (MA) is smaller and the MD is longer than when the odor cue represents a large object. This is due to the hand grip adjusted by visual feedback at the later stages of execution processing (Tubaldi et al., 2009). Interestingly, fruit juice can also make the same result when grasping a large target (Parma et al., 2011). These indicate that people can adjust their hand movement by olfactory and chemical senses. Using transcranial magnetic stimulation, researchers found that the participants’ hand motor potential is increased when the odor of food is congruent with the target (Rossi et al., 2008).

Multisensory integration shows that individual can use lots of sensory information such as visual and auditory information to perceive (Ernst and Bülthoff, 2004). The integration of visual and olfactory information also exists and affects how to adjust movement. The odor cues can evoke a perceptual change when a human is judging an unclear movement of a target. However, the aforementioned studies are narrow in focus in that they examine only the effects of olfactory information on the motor system; in addition, the results of these studies have led the authors to incongruent conclusions. On the one hand, olfactory information really affects a reach-to-grasp planning on the basis of our review such as reaction time (RT) and also affects execution such as MD. On the other hand, an interaction between visual and olfactory systems has yet to be clear, and it is unknown how interactions affect hand movement. To advance the understanding of multisensory integration and optimization of movement performance, the purpose of the present investigation was to explore the integration between visual and olfactory stimuli on movement. If a preceding olfactory information influences a human collecting information about a target, then there should be an effect on hand movement kinematics that should be measurable. We hypothesized that a reach-to-grasp hand movement would be enhanced when the odor cue was congruent with the visual target. The olfactory information provides right cues to make a movement plan, and the visual feedback may help us to control actions. That promotes participants’ RT and MD. By contrast, if the size of odor-cued object (OCO) and visual target were incongruent, interference effects would exist. Participants may make the wrong plan and waste time to adjust to the execution. We furthermore hypothesized that visual feedback can help them to correct the movement error. Thus, the specific aims of present study were to explore the effects of olfactory information on the execution of the reach-to-grasp movement and to compare the behavioral differences associated with visuo-olfaction integration.

## Materials and Methods

### Participants

In total, 29 young adults (15 women and 14 men) between 18 and 25 years of age (mean, 23.03 ± 1.69 years) participated in this experiment. Before starting the experiment, all participants answered a questionnaire about their history of nasal disease, smoking, exercise, and previous subjective status of olfactory function. All participants reported normal or corrected-to-normal vision as well as normal smell abilities, and all were right-handed. All eligible participants underwent the Sniffin’ Sticks test, and all showed good olfactory function and odor recognition. This test consists of 16 standardized odor pens (Burghart Messtechnik Company, Germany) that are presented to the participants. Participants identify the smell using a forced choice of four alternatives (one is correct and three are distractors). Each pen is held approximately 2 cm away from the nose. The interval between presentation of the odor pens is at least 30 s (Hummel et al., 1997).

Participants were naive to the purpose of the present experiment. Each participant’s role in the experiment was approximately 1.5 h. Participants were financially compensated for their participation once they had completed the experiment. The experimental procedures were conducted in accordance with the recommendations of the ethics committee of the Sport of Psychology Department at Shanghai University of Sport, which approved the protocol for this study. Written informed consent was obtained from each participant in accordance with the Sport of Psychology Department at Shanghai University of Sport.

### Apparatus and Stimuli

In consideration of ecological validity, the targets consisted of four real fruits or food that were grouped based on their relative sizes: small (garlic and ginger) and large (apple and orange). The targets were selected so that their visual features and size would be similar throughout the experimental period. The reach-to-grasp motion of the small targets required a precision grip, with the thumb pressed in opposition to the index finger. The large targets required a power grip, with the fingers flexed against the palm. The odor stimuli were congruent with the targets, that is, ginger, garlic, apple, and orange. The ginger odor was selected from the extended identification test, and others were from the original Sniffin’ Sticks (Hummel et al., 1997; Jessica et al., 2012). The scents were delivered by placing the odor stick approximately 2 cm from both nostrils of each participant in a well-ventilated room (Burghart Messtechnik Company, Germany).

Vision was controlled using a liquid glass (polymer-dispersed liquid-crystal) screen that rendered the target visually accessible by changing from translucent to transparent in 1 ms. We set an infrared automatic sensor switch to change the liquid glass. It changed the liquid glass from opaque to transparent, which represents a start signal. Visual feedback was provided under two conditions: open loop and closed loop. For the open-loop condition, participants received no visual feedback, whereas in the closed-loop condition, they were provided visual feedback. According to planning-control model, the entire grasping process consists of two stages: motor planning and online controlling. The grasping aperture influenced by the size of OCO mainly occurs in the previous stage. As the grasping movement progressed, visual feedback in a closed loop corrected the hand performance. But in an open loop, the purpose of isolated vision was to remove the online correction, so as to investigate the impact of the size of OCO modulate hand movement.

**FIGURE 1:**
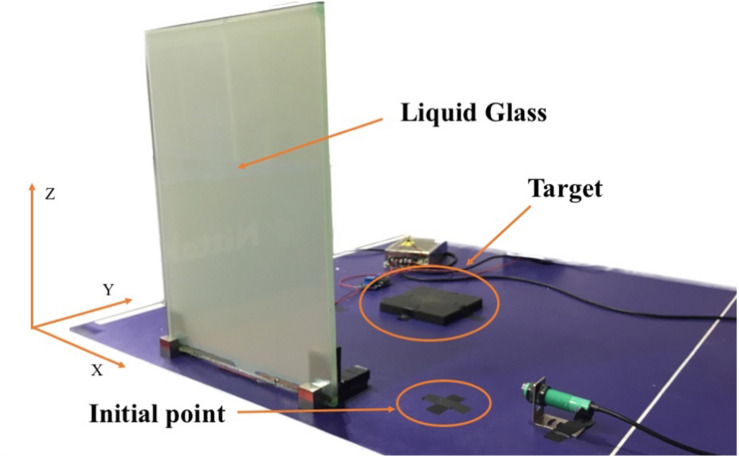
Image of the experimental setup.

The target to be grasped was placed along the midline of the participant’s body and 20 cm away from an initial point on the laboratory bench (see Figure 1).

Movements were recorded by a Simi Motion capture system equipped with four high-speed cameras and a shooting frequency of 100 Hz (Simi Reality Motion Systems GmbH, Germany). During experimental preparation, a T-wand and an L-frame were used to create a valid three-dimensional (3-D) calibration as a reference system for measurements. The T-wand was used to apply a known size to the system for metric calibration, and we moved it around the capture space for 30 s at least. The L-frame with two different shanks was aligned at the laboratory bench. The long shank indicated the *Y*-axis, and the short shank indicated the *X*-axis. Then we tracked the movement videos for calibration. The *Z*-axis was perpendicular to both axes (see Figure 1). The kinematics data were collected based on the position of three markers, each with a diameter of 14 mm. One marker was attached to a participant’s right hand between the thumb and the index finger, the second marker at the tip of the index finger, and the third marker at the tip of the thumb. Data processing and analyses were conducted using Simi Motion software.

### Procedure and Design

This study was a conducted using a within-subjects design. The odor cues comprised the following three levels: congruent with the size of the target, incongruent with the size of the target, and an odorless air control. The visual feedback had two levels: open loop (no visual feedback) and closed loop (visual feedback presented). Open loop refers to a control system in which the input signal of the system is not affected by the output signal. That is, the results of the control are not fed back into the system under control. After a start signal is received, the liquid glass will turn to opaque immediately, and participants will lose visual feedback and be unable to see their hand during grasping movement. A closed-loop system, also known as a feedback control system, compares the output with the expected value to determine any deviation. The liquid glass will not be opaque, and participants can see their hand movement. This deviation is then used to adjust and control the output value to make it as close as possible to the expected value. The combinations of the targets and odor stimuli were performed under six experimental conditions: (1) congruent small—one of the small targets was paired with the odor of one of the small objects; (2) congruent large—one of the large targets was paired with the odor of one of the large objects; (3) incongruent small—the odor from one of the large objects is paired with a smaller target; (4) incongruent large—the odor from one of the small objects was paired with a larger target object; (5) small control—participants reached to grasp a small object after presentation of odorless air; and (6) large control—participants reached to grasp a large target after presentation of odorless air (Table 1).

**TABLE 1:**
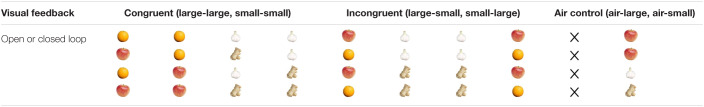
Combinations of odor cues and visual feedback.

*The left side of each column represents an odor cue, and the right side of each column represents the target to be grasped.*

**FIGURE 2:**
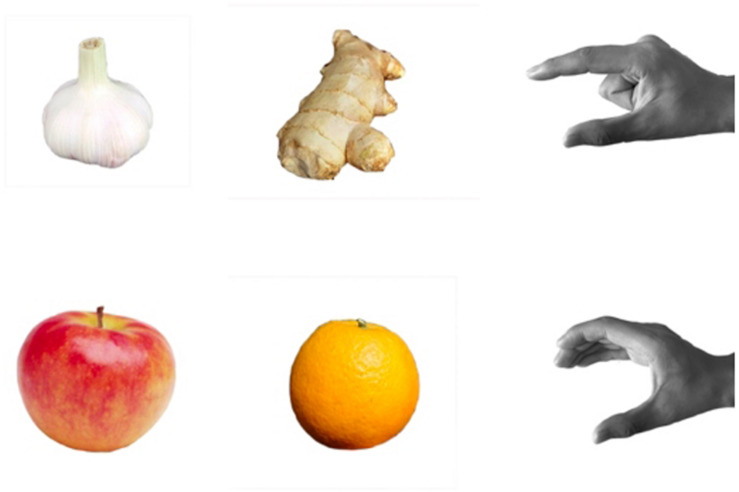
Target and hand grip gestures. The first row represents a precision grip for a small target, whereas the second row represents a power grip for the larger target.

At the beginning of each trial, participants’ right hand fingers closed together and placed their right hands on the initial position at the laboratory bench, and they placed their forearms horizontally on the laboratory bench. Before the experiment began, an investigator showed each target to be grasped and demonstrated the correct right-hand grip gesture for each target (Figure 2). Participants were verbally instructed not to grasp a target by the stem.

**FIGURE 3:**
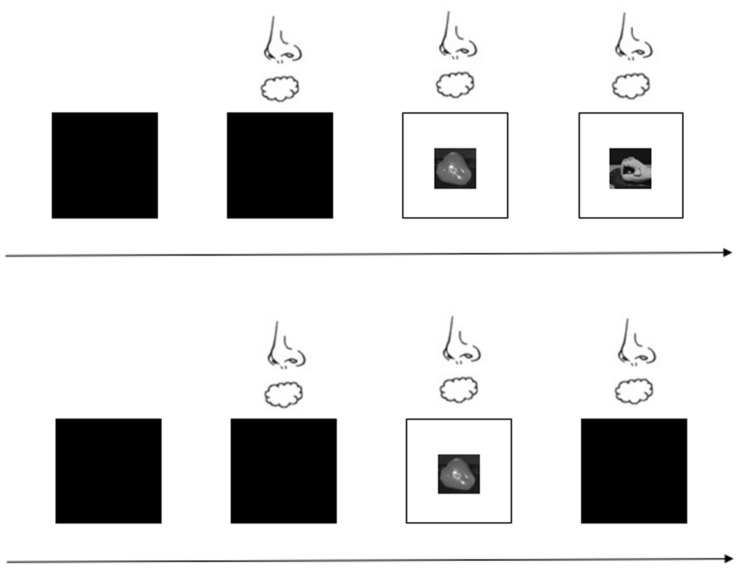
Experimental procedure with visual feedback provided or blocked. The first row represents the closed-loop condition; that is, visual feedback is provided. The second row represents the open-loop condition; that is, no visual feedback is provided.

For the formal experiment, participants performed a total of 80 trials presented in two blocks (40 trials for the open-loop condition and 40 trials for the closed-loop condition). The trial types were randomized within and across participants. Before a target was placed on the laboratory bench, the liquid glass was translucent so participants could not see the target. After the investigator set the target, the odor or odorless air was delivered to the participant for approximately 2 s, after which the liquid glass turned transparent so that participants could see the target. This clearing signaled participants to reach for the target and grasp it as quickly as possible. However, the moment the participants reached toward the target, the liquid glass either remained clear for the closed-loop condition or turned translucent again within 1 ms so that they no longer saw the target in the open-loop condition. Participants grasped and lifted the target using the hand grasp movement that had been demonstrated for that target. Figure 3 shows the experiment under the open- and closed-loop conditions. The time between trials was 10 s, which is sufficient to recover from odor adaptation (Hummel et al., 1997), and participants were allowed to rest or drink water during this time. The experimenter monitored each trial to ensure participant compliance with the requirements. In addition, the participants were asked to identify the odor in a randomly selected 10% of trials.

### Data Recording and Statistical Analysis

The reaching and grasping movement was divided into two phases: the planning process, in which participants estimated the size of the target and selected a correct hand grip gesture, and the actual execution process. Before experiment, the liquid glass was translucent; thus, participants could not see the target. After the experiment began, the liquid glass became transparent. The moment the glass cleared, the target appeared, as represented on the video recording as *n*_1_. When the velocity of the mark placed on the right hand first reached 20 mm/s, that moment on the video recording was considered *n*_2_ and represented the start of the movement. When the velocity of thumb and index finger decreased to 40 mm/s, that moment on the video recording was considered *n*_4_ and represented the end of the movement (Whitwell et al., 2008). The RT was calculated by subtracting *n*_1_ from *n*_2_ (i.e., *n*_2_ - *n*_1_), and the MD was calculated by subtracting *n*_2_ from *n*_4_ (i.e., *n*_4_ - *n*_2_). The raw data were smoothed by low-pass filtering (10 Hz) and obtaining the 3-D coordinate values of all markers (Chiou et al., 2012). The MA during the reaching and grasping task was calculated by the square root of [(*x*_2_ - x_1_)^2^ + (*y*_2_ - *y*_1_)^2^ + (*z*_2_ - *z*_1_)^2^], and that moment of the video recording was considered *n*_3_. The time to reach the MA (MAT) was calculated by subtracting *n*_2_ from *n*_3_ (i.e., *n*_3_ - *n*_2_). In our experiments, participants did not start hand movement in the planning process, so the RT is a measure of movement plan. In the execution process, participants did actual hand movement; and we collect MD, MA, and MAT to measure execution results. To assess the effects of the odor stimuli on the hand movements, repeated-measures analyses of variance (ANOVAs) were performed. Greenhouse–Geisser correction was applied to the degrees of freedom of *F* statistics when the Mauchly test showed that the sphericity assumption was violated (*p* < 0.05). To investigate the separated grasping type, paired-sample *t* tests were performed to determine whether there was a significant difference in results.

## Results

For the analyses, the independent variables were the odor cues and the visual feedback, and the dependent variables were the RT, MD, MA, and MAT.

### Reaction Time

**FIGURE 4:**
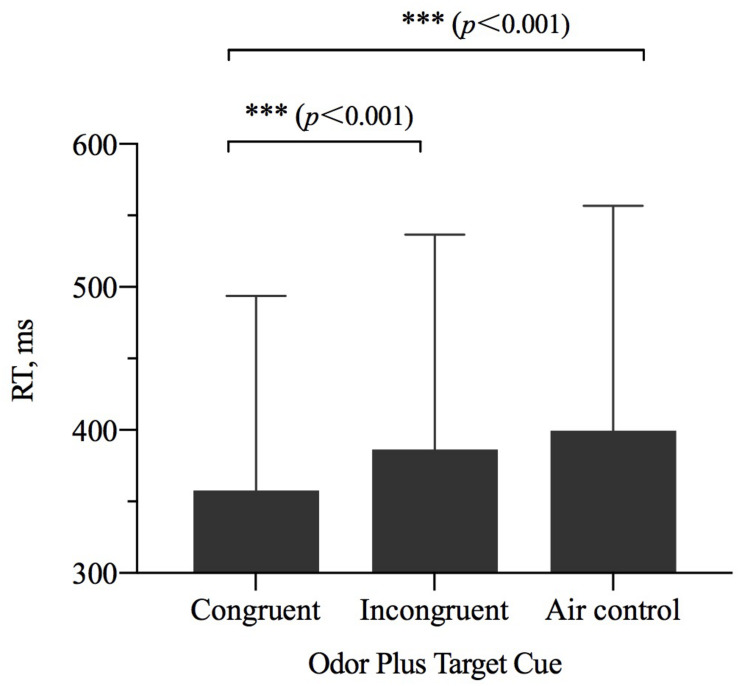
The reaction time (RT, *M* ± SD) for the combinations of odor cue and target size and visibility conditions. Error bars indicate SE. ^∗∗∗^*p* < 0.001 for the indicated comparisons.

The results of repeated-measures ANOVAs examining RT showed a significant main effect of odor cue [η^2^_p_ = 0.342, *F*_(2,27__)_ = 14.584, *p* < 0.001] (Table 2). The results of a pairwise comparisons indicated that when the size of OCO was congruent with the size of target, participant RT was faster than when the size of OCO was incongruent with the size of target (*p* < 0.001) or when the odorless air control was used (*p* < 0.001) (Figure 4).

**TABLE 2:** Reaction time for the various conditions (*M* ± SD, ms).

	Congruent odor and target	Incongruent odor and target	Odorless air control
	357.71 ± 136.16	386.34 ± 150.37	399.52 ± 157.28
	**Odor-target**		
Power grip	Congruent	357.59 ± 143.71	
	Incongruent	378.63 ± 156.38	
	Odorless air	391.93 ± 158.66	
Precision grip	Congruent	358.71 ± 133.32	
	Incongruent	389.36 ± 152.60	
	Odorless air	405.62 ± 168.65	

The two cases of RT were analyzed separately with ANOVAs. There was a significance main effect of odor cue [precision grip: η^2^_p_ = 0.212, *F*_(2,56)_ = 7.540, *p* = 0.001; power grip: η^2^_p_ = 0.200, *F*_(2,56)_ = 7.002, *p* = 0.002]. In the precision grip condition, the congruent size of OCO was different from the incongruent size of OCO (*p* = 0.001) and air control group (*p* < 0.001). In the power grip condition, the congruent size of OCO was different from that of the other two groups (incongruent group: *p* = 0.017; air control group: *p* = 0.001).

### Movement Duration

**FIGURE 5:**
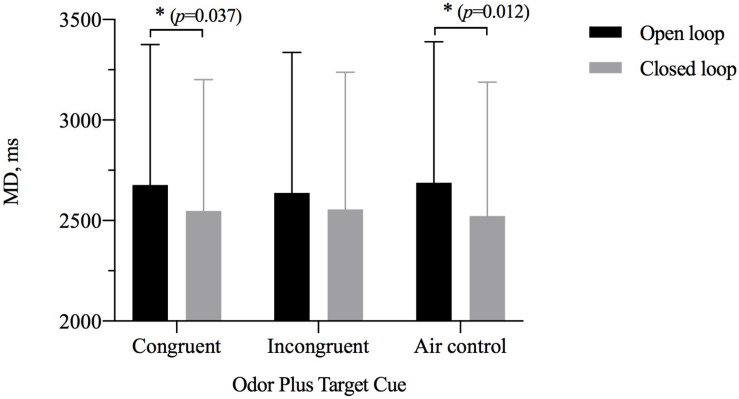
The movement duration (MD, *M* ± SD) for odor cue and target size and visibility combinations. Error bars indicate mean SE. ^∗^*p* < 0.05 for the indicated comparisons.

The results of repeated-measures ANOVAs assessing MD showed a significant main effect of visual feedback [η^2^_p_ = 0.150, *F*_(1,28)_ = 4.392, *p* = 0.035) but not of odor cue [η^2^_p_ = 0.027, *F*_(2,27)_ = 0.429, *p* = 0.653] and significant interaction between the two [η^2^_p_ = 0.198, *F*_(2,27)_ = 3.832, *p* = 0.028] (Table 3). A simple-effects analysis showed that the MD for the closed-loop condition was shorter than that for the open-loop condition when participants were exposed to an odor with a size congruent with the target (*p* = 0.037) or when they were exposed to odorless air (*p* = 0.012). However, there was no significant difference between the open- and closed-loop conditions for the size of OCO incongruent with the size of the target (Figure 5). The MD of two grip style was analyzed separately with ANOVAs. In the precision grip condition, the results showed a significant main effect of visual feedback [η^2^_p_ = 0.183, *F*_(1,28)_ = 6.288, *p* = 0.188] and odor cue [η^2^_p_ = 0.454, *F*_(2,27)_ = 11.228, *p* < 0.001]. The congruent size of OCO was different from the incongruent size of OCO (*p* = 0.024) and air control group (*p* < 0.001). In the power grip condition, the results showed a significant main effect of visual feedback [η^2^_p_ = 0.166, *F*_(1,28)_ = 5.585, *p* = 0.025] and odor cue [η^2^_p_ = 0.227, *F*_(2,27)_ = 3.957, *p* = 0.031]. The incongruent size of OCO was different from air control group (*p* = 0.008).

**TABLE 3:** Movement duration for the various conditions (*M* ± SD, ms).

Visual feedback	Congruent odor and target	Incongruent odor and target		Odorless air control
Open loop	2,676.66 ± 698.78	2,637.90 ± 698.06		2,687.45 ± 701.69
Closed loop	2,547.59 ± 653.42	2,555.90 ± 682.07		2,522.31 ± 665.72

	**Open loop**		**Closed loop**	

	**Odor-target**		**Odor-target**	
Power grip	Congruent	2,692.22 ± 683.18	Congruent	2,593.13 ± 647.96
	Incongruent	2,640.72 ± 716.48	Incongruent	2,581.32 ± 701.42
	Odorless air	2,759.05 ± 687.87	Odorless air	2,596.26 ± 675.58
Precision grip	Congruent	2,661.30 ± 726.33	Congruent	2,581.32 ± 701.42
	Incongruent	2,629.72 ± 703.33	Incongruent	2,513.67 ± 657.05
	Odorless air	2,607.47 ± 740.65	Odorless air	2,459.73 ± 657.24

The results of paired-samples *t* tests showed significant interaction. When grasping large target, MD in the closed loop was faster than in open loop [large odor condition: *t*(28) = 2.354, Cohen’s *d* = 0.44, *p* = 0.026; air condition: *t*(28) = 2.756, Cohen’s *d* = 0.51, *p* = 0.010]. When grasping small target, MD in open loop was slower than in the closed loop [large odor condition: *t*(28) = 2.569, Cohen’s *d* = 0.48, *p* = 0.016; air condition: *t*(28) = 3.059, Cohen’s *d* = 0.57, *p* = 0.005]. But in the closed loop, power grip’s MD for the small odor was faster than in the air condition [*t*(28) = −4.042, Cohen’s *d* = 0.75, *p* < 0.001]. Precision grip’s MD in the air condition was slower than in the small odor condition and in the large odor condition [small odor: *t*(28) = 5.031, Cohen’s *d* = 0.93, *p* < 0.001; large odor: *t*(28) = 2.788, Cohen’s *d* = 0.52, *p* = 0.009].

### Maximum Aperture

The results of repeated-measures ANOVAs examining MA indicated that there was no significant main effect of visual feedback or of odor cue [η^2^_p_ = 0.012, *F*_(1,28)_ = 0.348, *p* = 0.560; η^2^_p_ = 0.055, *F*_(2,27)_ = 0.568, *p* = 0.570] and no interaction between the two main effects [η^2^_p_ = 0.071, *F*_(2,27)_ = 0.841, *p* = 0.437] (Table 4). We also analyzed the results separately by grip kinds with ANOVAs. In the precision grip condition, the results did not show a significant main effect of visual feedback [η^2^_p_ = 0.330, *F*_(1,28)_ = 0.984, *p* = 0.330] and odor cue [η^2^_p_ = 0.103, *F*_(2,27)_ = 1.546, *p* = 0.231]. In the power grip condition, the results did not show a significant main effect of visual feedback [η^2^_p_ = 0.130, *F*_(1,28)_ = 4.195, *p* = 0.050] and odor cue [η^2^_p_ = 0.035, *F*_(2,27)_ = 0.485, *p* = 0.621].

**TABLE 4:** Maximum aperture for the various conditions (*M* ± SD, mm).

Visual feedback	Congruent odor and target	Incongruent odor and target		Odorless air control
Open loop	119.20 ± 14.95	120.86 ± 17.77		121.05 ± 13.32
Closed loop	122.18 ± 15.59	123.13 ± 18.25		121.08 ± 15.22

	**Open loop**		**Closed loop**	

	**Odor-target**		**Odor-target**	
Power grip	Congruent	121.47 ± 17.40	Congruent	127.88 ± 19.20
	Incongruent	123.22 ± 18.35	Incongruent	127.02 ± 17.35
	Odorless air	122.69 ± 17.33	Odorless air	123.88 ± 17.84
Precision grip	Congruent	116.76 ± 20.13	Congruent	116.75 ± 19.28
	Incongruent	117.61 ± 21.90	Incongruent	120.75 ± 22.10
	Odorless air	118.35 ± 18.66	Odorless air	119.26 ± 22.68

But the pair-samples *t* tests showed that precision grip’s MA in the small odor condition was smaller than in the large odor condition [*t*(28) = −2.283, Cohen’s *d* = 0.42, *p* = 0.030]. Power grip’s MA in the closed loop was larger than in open loop [large odor condition: *t*(28) = −2.620, Cohen’s *d* = 0.49, *p* = 0.014].

### Time to Reach the Maximum Aperture

**FIGURE 6:**
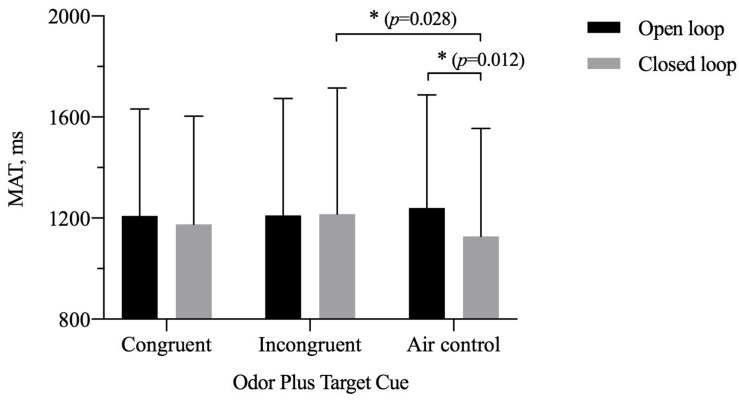
The time to maximum aperture (MAT, *M* ± SD) for odor cue and target size and visibility combinations. Error bars indicate mean SE. ^∗^*p* < 0.05 for the indicated comparisons.

The results of repeated-measures ANOVAs examining MAT showed a significant interaction between odor cue and visual feedback [η^2^_p_ = 0.057, *F*_(2,27)_ = 4.367, *p* = 0.017], but there was no significant main effect of visual feedback [η^2^_p_ = = 0.068, *F*_(1,28)_ = 2.032, *p* = 0.165] or of odor cue [η^2^_p_ = 0.267, *F*_(2,27)_ = 0.893, *p* = 0.031] (Table 5). As shown in Figure 6, a simple-effects analysis indicated that the MAT for the closed-loop condition was different when participants were exposed to an odor from an object incongruent with the size of the target compared with when they were exposed to odorless air. The MAT for the group exposed to an odor from an object incongruent with the size of the target was later than that of the odorless air control group (*p* = 0.028). Only for the odorless air control group was the MAT for the open-loop condition later than for the closed-loop condition (*p* = 0.012). In the precision grip condition, the results did not show a significant main effect of visual feedback [η^2^_p_ = 0.059, *F*_(1,28)_ = 1.761, *p* = 0.195] and odor cue [η^2^_p_ = 0.059, *F*_(2,27)_ = 1.786, *p* = 0.187]. In the power grip condition, the results did not show a significant main effect of visual feedback [η^2^_p_ = 0.069, *F*_(1,28)_ = 2.084, *p* = 0.160] and odor cue [η^2^_p_ = 0.101, *F*_(2,27)_ = 1.523, *p* = 0.236].

**TABLE 5:** Maximum aperture time for the various conditions (*M* ± SD, ms).

Visual feedback	Congruent odor and target	Incongruent odor and target		Odorless air control
Open loop	1,208.52 ± 423.13	1,210.45 ± 462.92		1,240.21 ± 447.62
Closed loop	1,175.10 ± 427.99	1,215.76 ± 499.61		1,127.21 ± 428.00

	**Open loop**		**Closed loop**	

	**Odor-target**		**Odor-target**	
Power grip	Congruent	1,233.59 ± 474.84	Congruent	1,200.76 ± 482.21
	Incongruent	1,199.03 ± 491.30	Incongruent	1,185.41 ± 510.56
	Odorless air	1,214.30 ± 470.09	Odorless air	1,073.13 ± 505.43
Precision grip	Congruent	1,183.67 ± 442.26	Congruent	1,149.91 ± 440.67
	Incongruent	1,203.14 ± 491.21	Incongruent	1,231.98 ± 566.65
	Odorless air	1,292.73 ± 529.65	Odorless air	1,171.55 ± 538.70

Pair-samples *t* tests indicated that visual feedback made a significant difference. Power grip’s MAT in the closed loop was early than in open loop [*t*(28) = 2.238, Cohen’s *d* = 0.41, *p* = 0.033]. It was early in the air condition than in the large odor condition and in the small odor condition [large odor: *t*(28) = 2.845, Cohen’s *d* = 0.53, *p* = 0.008; small odor: *t*(28) = 2.292, Cohen’s *d* = 0.46, *p* = 0.030]. Precision grip’s MAT in the small odor condition was earlier than in the air condition [*t*(28) = −2.061, Cohen’s *d* = 0.38, *p* = 0.049].

## Discussion

The principal goal of the present study was to advance the understanding of multisensory integration and optimization of movement performance by assessing the integration between visual and olfactory stimuli. To that end, we collected kinematics data from participants who were exposed to scents from objects that were the same size (congruent condition) or smaller or larger (incongruent conditions) than a target to be grasped, which was either seen or unseen as the participant completed the reach-to-grasp hand movement. The reaching and grasping movements were divided into the planning process, in which participants estimated the size of the target and selected a correct hand grip gesture, and the actual execution process. Our main finding was that there was an association between olfactory information and hand movements. When the size of OCO coincided with the size of a target to be grasped, the RT was optimized and significantly decreased. We also found that visual feedback enhanced MD in the congruent condition and in the odorless air condition but not in the incongruent condition. Although the amplitude of grip aperture might also have been disrupted in the nonvisible condition, our data could not demonstrate this.

In the planning process, the RT for the congruent condition was faster than that for the incongruent condition or for when odorless air was used as a control. A plausible explanation for this finding is that participants used the olfactory information to make an estimation of the ensuing hand movement. Thus, for a congruent condition, participants would select the correct movement plan and could immediately execute the action with no adjustment time necessary. When the size of OCO was incongruent with the visual target, however, participants had to adjust their incorrect movement decision, making the overall RT longer. In the execution phase, participant movement was faster not only in the congruent condition but also in the odorless air condition when participants could see the target. However, even with visual feedback, incongruent odor/target cues might affect movement behavior. When visual information and olfactory information are presented at the same time, both might influence the motor system, with the integrated visual and olfactory information promoting recognition of the target. Once such an interaction occurs, it may increase the activation of the right middle temporal cortex and the left superior temporal cortex. Increased activation of these brain regions is related to vision and olfaction and provides evidence of a brain network for the interaction (Tubaldi et al., 2011).

The planning-control model holds that humans use cues in the environment to generate motor plans, but the correction and control of the ensuing movements in the execution process are not affected by these environmental cues (Glover and Dixon, 2001). Before movement begins, humans receive information from the environment as clues to initiate appropriate motor plans. After the movement begins, the outcomes may still be modulated. In reality, vision and olfactory typically coexist. However, the present findings suggested that olfactory information can affect motor planning even in the presence of visual feedback. Thus, odor can enrich the expression of motor information and coordinate with the effect of vision on movement (Tubaldi et al., 2008). *A priori* visual knowledge of a target cannot totally remove the interference generated from an inaccurate odor cue, but the brain has sufficient time to block the influence of useless or wrong odor cues and make a good final movement plan (Tubaldi et al., 2009). Olfactory information affected the hand movements based on present research. Even if the action plan was wrong, all of the participants wasted more times and aperture, and they could still grasp target by correct hand types. This may explain why participants in the present study all performed the right grip gesture during the experiments. When presented with an odor cue from a small object to grasp large target, the aperture of the grip could have been small, more a precision than a power grip, because of the influence of the odor cue; however, our results did not show a significant difference in the maximum size of the grip aperture. This finding may be because participants in the present study knew they had to consider the possibility of a power grip and were thus primed to immediately change their grip.

Different from the past, we investigated how the multisensory affect hand movements in planning and execution process. The open-loop experiments showed that when the size of target was congruent with the odor cues, the RT was decreased. These results support the idea that odor cues affect the planning process. Congruent with the inverse effectiveness theory (Stein and Stanford, 2008), our results indicated that when one sensory signal (vision, in this case) was decreased, olfactory information modulated movement. In addition, when visual information was blocked, congruent odor cues optimized processing information to shorten the RT. Thus, olfactory information can modulate the planning and execution of hand movements. Not only is such an effect true for hand movement, but also it has been shown for mouth movement. For example, the smell of food adjusts the size of the opening of the mouth (Parma et al., 2011). In addition, among children with autism, the odor of their mother activates faster reactions than the odor of a stranger (Parma et al., 2013). In our experiment, participants did hand movements in all conditions, and the task was easy to practice. When visual and olfactory information was presented at the same time, the integration promoted individual’s action recognition. The right side of middle temporal gyrus and the left side of superior parietal cortex activated higher in the integrated system. These areas were related to vision and olfactory (Tubaldi et al., 2011). Despite such results, the real fruit or food was grasped in our experiment, which resulted in errors inevitably. We did not use the brain-imaging technology, so the mechanisms underlying the ability of olfaction to affect reaching and grasping movements remain unknown.

## Conclusion

The results of the present work suggested that olfactory information influence reach-to-grasp kinematics. More specifically, preceding odors from objects congruent with the size of ensuing target motions generated positive influences on hand movements. In addition, our results supported an integration between vision and olfaction and suggested that integration of these sensory modalities can optimize motor behavior. To construct the integration theory of visual and olfactory information and motor sensory system, future research should investigate more clearly how the various sensory information affects hand movement.

## Data Availability Statement

The datasets generated for this study are available on request to the corresponding author.

## Ethics Statement

The studies involving human participants were reviewed and approved by School of Psychology Department at Shanghai University of Sport. The patients/participants provided their written informed consent to participate in this study.

## Author Contributions

XW and YY designed the study, conducted the experiments, wrote the manuscript and contributed to manuscript revision, and read and approved the submitted version. YY organized the database and analyzed the data.

## Conflict of Interest

The authors declare that the research was conducted in the absence of any commercial or financial relationships that could be construed as a potential conflict of interest.
